# Comparative insights into clinic onboarding and interaction practices for patient engagement in long COVID digital health care

**DOI:** 10.1177/20552076241294101

**Published:** 2024-11-26

**Authors:** Hadiza Ismaila, Ann Blandford, David Sunkersing, Fiona Stevenson, Henry Goodfellow

**Affiliations:** 1 Department of Primary Care and Population Health, 4919 University College London, London, UK; 2 573973 Department of Computer Science, UCLIC, University College London, London, UK; 3 Institute of Health Informatics, 4919 University College London, London, UK; 4 Department of Primary Care and Population Health, 4919 University College London, London, UK

**Keywords:** Mixed methods < studies, chronic < disease, digital health < general, telehealth < general, remote patient monitoring < personalised medicine, public health < disease, apps < personalised medicine

## Abstract

**Objectives:**

This study explores the influence of clinic-led onboarding and interaction processes on patient engagement within a digital health program for long COVID care, the Living with COVID Recovery (LWCR) program. We compared clinical practices and patient engagement levels across seven clinics utilising LWCR, gaining insights that could optimise digital health interventions (DHIs) for long COVID care.

**Methods:**

Using a mixed-methods approach, we conducted qualitative interviews with clinicians from seven clinics (out of thirty five) to understand their onboarding and interaction strategies for the LWCR program. We also performed a descriptive quantitative analysis of patient and clinic usage data to rank and classify patient engagement. These rankings were then compared against qualitative insights to explore the influence of clinic-led strategies on patient engagement. Additionally, we conducted interviews with 12 patients under the care of seven clinics to complement our mixed-method analysis.

**Results:**

Four key practices were identified in clinics with higher patient engagement: pre-assessment onboarding, proactive communication, patient education and the involvement of clinically experienced staff. Clinics that integrated these interdependent practices into a cohesive strategy demonstrated notably higher patient engagement levels.

**Conclusion:**

This study highlights the critical role of integrating multiple, interdependent clinic-led strategies to optimise patient engagement in DHIs, particularly for long COVID care. These findings provide actionable insights for healthcare providers and policymakers, suggesting that a comprehensive approach is essential for maximising patient engagement and improving care outcomes. The study lays the groundwork for future research to explore the broader applicability of these strategies across different healthcare contexts.

## Background 

### Healthcare challenges of long COVID

The COVID-19 pandemic strained healthcare systems worldwide. As these systems contended with acute COVID-19 cases, the long-term consequences of the virus, known as “long COVID” or post-acute sequelae of SARS-CoV-2 infection, emerged as a global health concern.^1^ Long COVID is characterised by persistent symptoms such as fatigue, breathlessness, cognitive impairments, muscle and joint pains and mental health issues such as anxiety and depression, lasting for months or years after the initial infection.^1^ A recent systematic review and meta-analysis spanning 41 studies indicated that approximately 43% of COVID-19 patients experience long COVID symptoms, although this prevalence varies across studies.^2^ The high prevalence and varied presentation of long COVID pose substantial challenges for healthcare systems worldwide, necessitating extensive clinical care, regular monitoring and prolonged rehabilitation for many affected individuals.^1^

In response to long COVID's growing impact, countries worldwide initiated dedicated care pathways to address its symptoms. The UK's National Health Service (NHS) has been at the forefront, establishing over 80 specialised long COVID clinics.^3^ These clinics deliver multidisciplinary care involving experts like pulmonologists, cardiologists, physiotherapists and mental health specialists.^3^ Despite these efforts, traditional care models face challenges such as limited in-person consultations due to pandemic protocols, resource constraints amidst rising long COVID cases and the varied nature of long COVID symptoms.^4^

### The integration of digital health interventions in long COVID care

In recent years, integrating digital health interventions (DHIs) into clinical care pathways for chronic disease management has become increasingly important.^5^ For instance, Von Theile Schwartz's concept of co-care emphasises the co-production of health outcomes through collaborative interactions between patients, healthcare providers and information and communication technology.^6^ This development has been particularly relevant in the context of long COVID due to the complexities of this emergent, chronic condition.^7^ Traditionally, DHIs have primarily focused on standalone self-management tools for established chronic conditions such as diabetes, hypertension and chronic obstructive pulmonary disease.^8^ However, with its broad spectrum of symptoms and unpredictable progression, long COVID necessitates a more integrated approach, where DHIs facilitate patient self-management and ensure continuous clinical oversight and support.^4,9^

The Living with COVID Recovery (LWCR) program, implemented across 35 UK clinics during this study, exemplifies this integrated care model. The program is designed to empower patients to manage their symptoms while providing clinicians with the tools to monitor patient progress and intervene when necessary. It comprises a patient-facing app, a clinical dashboard and a patient care pathway. This dual-purpose system facilitates direct communication and information sharing between patients and clinicians, fostering a dynamic and collaborative care environment. However, like many DHIs, successfully implementing LWCR into the long COVID care pathway depends on its effective uptake and ongoing patient engagement.^10^ This paper explores how various clinics have engaged patients with LWCR as part of their routine clinical practice. We examine how clinic-led onboarding processes and clinical interactions within LWCR may influence patient engagement. This is crucial for understanding the optimal implementation of DHIs in clinical settings, especially in enhancing patient engagement in long COVID care.

### Related work: clinic-led onboarding and interaction for patient engagement

Integrating DHIs into clinical care, particularly for complex conditions like long COVID, highlights the need for effective clinic-led onboarding and ongoing interaction strategies in fostering patient engagement. In the context of DHIs, ‘clinic-led onboarding’ refers to the process whereby healthcare providers introduce patients to DHIs, guiding them in understanding and effectively utilising these tools.^11^ This initial engagement is crucial as it sets the tone for patients’ future interactions and trust in the system.^11^ Meanwhile, ongoing interaction encompasses proactive and responsive communication between healthcare providers and patients through the DHI platform, crucial for maintaining patient involvement and responding to their evolving needs.^12^ This study addresses a significant gap in the literature by examining how healthcare providers influence patient engagement through clinic-led strategies.

Most existing studies focus on patient and technology-centric factors, often overlooking specific roles healthcare providers play in fostering this engagement.^13^ Studies that highlight the role of healthcare providers often lack specificity and depth in examining how clinic-led strategies directly impact patient engagement. For instance, a recent study conducted in Germany emphasised the importance of healthcare providers in enhancing patient access and adherence to digital therapeutics.^14^ However, it failed to examine specific strategies healthcare providers employ during onboarding and monitoring and how they are integrated into routine clinical care. Similarly, another study on patient-centred communication strategies used in a mHealth diabetes prevention program for older adults suggested that tailored, data-driven coaching and continuous support through in-app chats can support patient self-management.^15^ However, the study does not explicitly explore how such communication strategies impact patient engagement. To address these limitations, this research provides a detailed analysis of the specific clinic-led strategies that successfully enhance patient engagement in long COVID care.

Methodologically, this study extends the approach of a prior study that implemented a DHI to support psychosis recovery (App4Independence—A4i), which combined qualitative interviews with healthcare professionals and patients with quantitative analysis of patient engagement metrics. The A4i study provided valuable foundational insights into patient engagement within a mental health context, highlighting that proactive onboarding and monitoring strategies, including the involvement of digital navigators, clinicians, peer support workers and tailored educational materials, significantly enhanced patient engagement.^16^

However, the complexities of long COVID care—such as variable symptoms, diverse patient demographics and the need for multidisciplinary treatment teams—demand strategies that account for broader clinical variabilities and more complex patient needs. The current study builds on the A4i study's approach, adapting it to the multifaceted context of long COVID care. By employing a mixed method approach to systematically examine the impact of onboarding and interaction strategies on patient engagement across various long COVID clinical sites, our research has generated actionable insights crucial for optimising patient engagement strategies in managing long COVID.

The rest of the paper is organised as follows: The Methods section details the mixed-methods approach, combining qualitative interviews with clinicians and patients, and quantitative engagement metrics, to explore clinic-led onboarding and interaction strategies. The Results section presents findings from the comparative analysis, highlighting key practices that enhance patient engagement in long COVID care. The Discussion interprets the research findings, highlighting how the identified clinic-led strategies can significantly improve patient engagement in long COVID care. Finally, the Conclusion summarises the study's contributions and suggests avenues for future research.

## Methods

We adopted a mixed-methods approach, combining in-depth qualitative interviews with descriptive quantitative engagement metrics, to explore the nuances of clinic-led onboarding and interaction strategies across various clinics and their association with patient engagement levels in a long COVID digital health context (Table 1).

**Table 1.** Clinic data for clinician interviews.

Clinic ID	Patients	Clinicians	Region	Clinician interview date	Clinician participants
C1	320	12	Southeast England	07 Jul 21	2
C2	792	22	Southeast England	28 Jul 21	1
C3	689	17	Southeast England	10 Sep 21	1
C4	184	9	London	19 Nov 21	4
C5	1350	37	Southeast England	24 Nov 21	1
C6	604	34	London	29 Mar 22	4
C7	160	9	East of England	06 Apr 2022	1

### Semi-structured interviews

#### Clinician interviews

We extended email invitations to clinicians across all 35 clinics implementing LWCR, securing participation from 14 clinicians across seven clinics. Where multiple clinicians from one clinic participated, they were interviewed together; the same semi-structured topic guide was used regardless of number of participants and the focus was on the shared perspectives of participants rather than probing differences. The third and last authors conducted semi-structured interviews between July 2021 and April 2022 to gain insights into how LWCR was integrated into clinical pathways. All clinician interviews were conducted remotely using secure video conferencing tools, including Zoom and Microsoft Teams, and lasted 30 to 45 min. Remote interviews accommodated the geographical spread of participants and adhered to the pandemic-related health guidelines during the study period. We obtained written informed consent from all participating clinicians before the interviews. The semi-structured interview guide covered various topics, including patient onboarding, engagement strategies with LWCR and the successes and challenges faced during implementation. The interviews were audio-recorded with participant consent, transcribed verbatim by a professional service and anonymised before data analysis. For analytical purposes, we treated responses from the same clinic as collective insights rather than individual input, ensuring a unified representation of each clinic's experience with LWCR (Table 2).

**Table 2.** Patient demographic information.

Interview date	ID	Gender	Patient supplied education	Ethnicity	Clinic ID	Region
28 Mar 22	B1	F	Degree or postgraduate	White	O4	London
05 Apr 22	A2	F	School-leaver	Asian/Asian British	O2	London
11 Apr 22	A3	F	Degree or postgraduate	White	O1	East of England
12 Apr 22	A4	F	Nvq levels	Asian/Asian British	C6	London
13 May 22	A5	F	Degree or postgraduate	White	C2	London
17 May 22	A6	F	School-leaver	White	C2	London
08 Jul 22	B7	F	School-leaver	White	O1	East of England
21 Jul 22	A8	F	N/A	N/A	O3	East Midlands
25 Jul 22	B9	F	School-leaver	White	C6	London
26 Jul 22	A10	M	Nvq levels	Other ethnic group	O2	London
12 Sep 22	A11	F	Nvq levels	White	C5	Southeast England
15 Sep 22	A12	F	School-leaver	White	O1	East of England

F: female; M: male; NVQ: national vocational qualification.

#### Patient interviews

In addition to clinician interviews, we sought patient perspectives to enrich our understanding of LWCR's role in clinical care. Throughout the project, anonymised clinic and patient information had been gathered into a data repository in Metabase (https://www.metabase.com/). Initially, we identified participants by accessing the Metabase repository to stratify users based on their engagement levels with the app and purposefully selected a representative sample across diverse demographics. Patients were then contacted via email, and those who agreed to participate confirmed their availability. Despite efforts to ensure diversity by inviting 239 patients across 35 clinics, only 12 patients from seven clinics—three overlapping with the clinician cohort—participated. This highlighted recruitment challenges and limited our scope for direct clinic-to-clinic comparison. The patient group was predominantly female (11 out of 12) and mostly white (eight out of 12), with educational backgrounds ranging from school leavers to postgraduates. We obtained written informed consent from all participating patients before the interviews.

The patient interviews were conducted remotely using Microsoft Teams between March 2022 and August 2022. The interview guide was semi-structured, covering topics such as onboarding experiences, interactions with clinics through the program and overall care experiences. Participation in the interviews was voluntary, with no compensation provided. Following each interview, audio recordings were transferred securely, transcribed by a professional service and stored anonymised for data analysis. Despite the recruitment challenges, the insights gathered from these patient interviews add depth to our understanding of clinician-reported strategies and their impact on clinical care.

The study was granted ethical clearance from the Health Research Authority East Midlands—Derby Research Ethics Committee on 23 July 2021 (research ethics committee (REC) reference: 21/EM/0160).

### Qualitative data analysis

#### Clinician interviews

In our analysis of clinician interviews, we utilised a comparative thematic approach^17^ to uncover the nuances of patient onboarding and ongoing interactions within the seven clinics involved. Our thematic analysis was inductive, allowing themes to be identified from the data without imposing a pre-existing theoretical framework. The data analysis process involved detailed line-by-line reading of interview transcripts, during which initial codes were applied to text segments using NVivo software. These codes were iteratively refined and grouped into broader categories that formed the basis of identified themes that captured the clinics’ strategies for engaging patients. Once potential themes were identified, they were reviewed in several iterative rounds to refine their definitions and ensure they accurately reflected the data. This process included revisiting the data excerpts associated with each theme, discussing their relevance with the research team and adjusting the thematic framework where necessary. The first author primarily conducted the coding and initial thematic analysis, with the identified themes subsequently reviewed and validated by both the second and last authors to ensure reliability and mitigate bias.

Finally, we conducted a comparative analysis, comparing the identified themes across the seven clinics. This process helped us identify both unique and shared strategies in patient onboarding and ongoing interaction. Due to the study's design, which focused on capturing a broad range of perspectives from clinicians across various clinics rather than achieving thematic saturation, we did not specifically test for data saturation.

#### Patient interviews

We thematically analysed patient narratives to supplement our clinician interview findings, focusing on their onboarding and ongoing interaction experiences with clinics. We thoroughly reviewed and coded the patient interview transcripts, highlighting instances where patient accounts aligned with, differed from or added new dimensions to the clinician-reported strategies. These patient insights were then integrated into relevant clinician-derived themes, allowing for a nuanced comparison of perspectives and enhancing the comprehensiveness of our findings.

### Quantitative data exploration

Throughout the project, anonymised data gathered through the LWCR platform was stored in a data warehouse, MetaBase, and made available to the research team. Concurrently with our qualitative analysis, we undertook a quantitative descriptive analysis of patient engagement data within the LWCR app, retrieved from the MetaBase database. This analysis focused exclusively on the seven clinics featured in the clinician interviews to evaluate their performance based on selected engagement metrics. This targeted approach allowed for a comparative analysis of engagement metrics against reported clinical practices from the same clinics.

#### Engagement metrics selection

Our analysis focused on metrics evaluating the impact of clinic-led onboarding and interactions in long COVID clinics. We prioritised metrics related to key app features such as patient sign-up, patient-reported outcome measures (PROM) questionnaires,^18^ and in-app messaging. To assess the effectiveness of onboarding, we examined registration rates, completion rates and the speeds of initial questionnaires within the first month after registration. While a feature of the LWCR program to assist in patient assessments, the PROM questionnaires were not utilised as data collection methods in this study. Instead, our analysis focused on measuring these questionnaires’ completion rates and response times to examine patient engagement with the program. Permission to use all applicable tools and questionnaires was obtained from the respective copyright holders. These metrics reflect immediate engagement, with registration representing the first system interaction and questionnaire completion, providing early patient assessments critical for care initiation.

Additionally, we evaluated the effects of clinic interaction strategies by examining the average number of questionnaires completed and messages sent per patient during the first three months, a period critical for active care management. These metrics help measure active engagement and responsiveness to clinic strategies. We further assessed how these metrics correlate with clinic message rates to understand their impact on patient engagement within this timeframe. Detailed tables of these metrics are provided below (Tables 3 and 4).

**Table 3.** Onboarding engagement metrics for the first month of using the app.

Metric category	Specific metrics	Explanation
Registration	Patient registration rate	The percentage of patients invited to use LWCR by a clinician that went on to complete the registration process.
PROMs questionnaire	Questionnaire completion rate	The percentage of patient who completed at least one questionnaire within the first month of using LWCR.
	Initial questionnaire completion speed	The average time it takes for patients to complete the initial questionnaire post registration.

LWCR: Living with COVID Recovery; PROM: patient-reported outcome measure.

**Table 4.** Clinical interaction metrics (within first 3 months post-registration).

Metric category	Specific metrics	Explanation
Patient engagement metric		
Questionnaire completion	Average questionnaires per patient	Average number of questionnaires completed per patient within the first 3 months of registration
Messaging	Average patient message per patient	Average number of patient messages sent per patient within the first 3 months of registration
Clinic engagement metric		
Messaging	Clinic message rate	Percentage of patients sent messages from the clinic

#### Quantitative data analysis

Our analytical approach was descriptive, utilising statistical package for the social sciences (SPSS) (Version 29). Given the multifaceted nature of patient engagement and the complexity of factors influencing it, we determined that inferential statistics were beyond the scope of this study. Instead, we focused on establishing performance-based classifications for the seven clinics, using statistical benchmarks for each engagement metric to identify and highlight best practices.

##### Performance classification framework

We employed a standardised classification framework based on quartile analysis to assess and compare clinic performance across various engagement metrics.^19^ This includes the first quartile (Q1: 25th percentile), the median (Q2: 50th percentile) and the third quartile (Q3: 75th percentile).^19^ These benchmarks enable a relative performance assessment, highlighting comparative strengths and weaknesses within our select group**.**

#### Criteria definition and application

We classified clinic performance into three tiers, reflecting their proximity to quartile benchmarks. With only seven clinics, dividing performance into three tiers – top, moderate and lower – allows for precise and meaningful differentiation without over-segmentation, which in a small sample might obscure actionable insights.

- Top performers (TP) (upper quartile): clinics whose performance measures meet or exceed Q3 indicate leadership and set performance benchmarks within the cohort.
- Moderate performers (TP) (mid-range): clinics with performance between Q1 and Q3, reflecting typical engagement levels relative to their peers.
- Lower performers (LP) (lower quartile): clinics performing at or below Q1, identifying those requiring targeted improvements to elevate their performance to the cohort's standard.

#### Adjustments based on proximity

For each metric analysed, we examined the proximity of Q1 and Q3 to the median and extreme values to determine if adjustments to classification boundaries are necessary. This ensures that our classification tiers accurately reflect each clinic's relative performance. This adjustment is especially crucial in small samples, where a strict quartile method might not accurately capture the actual differences in performance. In Results section, we provide justifications for any adjustments made to classification thresholds based on the proximity of quartiles to the median and extremes, ensuring a nuanced approach to performance evaluation.

### Mixed-methods comparative analysis

Our mixed-methods comparative analysis systematically aligned the qualitative themes with the quantitative performance data from corresponding clinics to uncover patterns of interdependency between the reported practices and patient engagement outcomes. By Integrating these two data sets, we identified specific practices associated with higher engagement and examined how these practices interacted to create more effective patient engagement strategies.

For example, we analysed clinics with high registration and questionnaire completion rates (quantitative metrics) to determine the qualitative strategies driving these outcomes. We then compared these strategies with those employed by clinics with lower engagement metrics. The integration process was iterative: quantitative findings guided the exploration of qualitative data, and insights from qualitative analysis informed the interpretation of quantitative outcomes. This dynamic interplay between data sets allowed us to identify the most effective practices and their interdependencies.

Furthermore, the integrated analysis revealed discrepancies between perceived and actual engagement outcomes. In some cases, clinics that believed they were implementing effective strategies did not show corresponding high engagement metrics. This prompted us to conduct a deeper qualitative investigation into potential external factors or implementation challenges, offering a more comprehensive understanding of the complexities involved in patient engagement.

## Results: comparative mixed-methods findings

In this section, we outline our findings in two distinct phases: first, we analyse quantitative metrics for initial engagement, including registration and questionnaire completion rates and speeds, and compare these with qualitative insights into onboarding processes. Next, we examine ongoing engagement metrics in clinic-patient interactions and their correlation with qualitative data on interaction strategies.

### Quantitative results: onboarding initial engagement metrics

Tables 5 to 7 detail descriptive quantitative findings, summarising clinics’ performances based on initial patient engagement metrics such as registration rates, questionnaire completion rates and speeds within the first month post-registration. For extensive tables and detailed performance classification, refer to the supplemental document.

**Table 5.** Onboarding initial engagement metrics rates by clinic.

Clinic #	Patients invited	Patients registered	Registration rate %		Questionnaire completion rate %		Initial questionnaire completion speed (days)	
C1	320	292	91.25	TP	87.68	TP	1.14	TP
C2	792	708	89.39	TP	89.96	TP	1.56	MP
C3	689	515	74.74	MP	76.22	MP	2.23	LP
C4	184	126	68.48	LP	78.99	MP	2.28	LP
C5	1350	1132	83.85	MP	85.74	TP	1.80	MP
C6	604	464	76.82	MP	75.94	MP	2.07	MP
C7	160	103	64.38	LP	71.29	LP	1.68	MP

LP: lower performers; MP: moderate performers; TP: top performers.

**Table 6.** Descriptive statistics.

Statistics	Mean	Median	*SD*	Minimum	Maximum	Range	IQR	Q1	Q3
Registration rate (%)	78.42	76.82	9.46	64.38	91.25	26.88	20.92	68.48	89.39
Questionnaire completion rate (%)	80.83	78.99	7.00	71.29	89.96	18.70	11.74	75.94	87.68
Initial questionnaire completion speed (days)	1.82	1.80	0.40	1.14	2.28	1.14	0.67	1.56	2.23

IQR: interquartile range.

**Table 7.** Overall performance ranking for onboarding initial engagement.

Clinic	RR	QCR	QCS	Total	Average	Rank	Classification
C1	3	3	3	9	3	1	Top performer
C2	3	3	2	8	2.67	2	Top performer
C5	2	3	2	7	2.33	3	Moderate performer
C6	2	2	2	6	2	4	Moderate performer
C3	2	2	1	5	1.67	5	Moderate performer
C7	1	1	2	4	1.33	6	Lower performer
C4	1	2	1	4	1.33	6	Lower performer

RR: Registration Rate; QCR: questionnaire completion rate; QCS: questionnaire completion speed.

#### Overall performance ranking and classification

To facilitate this analysis, we converted performance categories into numerical values to calculate overall rankings: TP: 3; MP: 2; LP: 1.

For overall classification, we categorised the clinics based on their average scores using the following thresholds:

- TPs: average scores >= 2.5
- MPs: scores between 2.5 and 1.5
- LPs: average scores =< 1.5 (closer to 1).

The clinics demonstrate varying performance metrics, with registration rates ranging from 64.38% to 91.25% and questionnaire completion rates from 71.29% to 89.96%. Initial questionnaire completion speed varies from 1.14 to 2.28 days. Based on these numbers:

Clinics C1 and C2 rank as TPs, with the highest registration and questionnaire completion rates and fastest completion speeds. Clinics C5, C6 and C3 are MPs, with Clinic C3 showing notably slower completion speeds. Clinics C4 and C7 are LPs, with the lowest registration and completion rates. Clinic C4 also exhibits slower questionnaire completion speeds.

### Comparative insights: onboarding process and initial engagement

This section examines the relationship between various clinics’ onboarding processes and their initial patient engagement outcomes. By comparing quantitative data on clinic performance in initial engagement metrics against qualitative insights, we identify patterns that could explain variations in clinic effectiveness.

#### Qualitative themes: onboarding process

Three key themes were identified based on the thematic analysis of the clinics’ onboarding process. These themes include:

1. The strategy implemented for introducing patients to LWCR.
2. The personnel involved in the onboarding process.
3. The timing of the onboarding.

##### Onboarding strategy (what)

Clinics employ various onboarding strategies, broadly categorised into comprehensive patient education or technical guidance. However, our analysis reveals no consistent relationship between the specific strategy used and the clinics’ performance in fostering initial patient engagement. This suggests that multiple factors may influence these outcomes.

#### Prioritising patient education

Clinics C1, C3, C4 and C6 prioritise comprehensive patient education to build trust and engagement. They focus on demonstrating the clinical utility of LWCR in securing patient buy-in. For example, a clinician at C4 highlighted the importance of informed initial discussions:

Having that really good conversation at the beginning, which speaking to somebody who has the knowledge, not only just about the functionality of the app but actually maybe to have an informed conversation about the actual clinical management, I think is huge and really important. And, again, I think that's about making people trust what you’re selling them and then helping them move into a place of being able to engage with it. C4.

Despite these efforts, clinic performance variations suggest subtle differences in educational approaches may influence engagement rates. Clinic C1 excels with a registration rate of 91.25% registration rate and a questionnaire completion rate of 87.68%. This success could partly be attributed to their follow-up strategies, as a clinician at C1 explains:

[The care coordinator] onboards patients onto the app; she sends them messages, she encourages them to fill out all their questionnaires […] what [the care coordinator] will do is she’ll look at their responses, and based on what their scores are, she’ll then send them a few suggestions of things that they might want to look at on the app to keep them engaged until we can get to see them. C1.

In contrast, Clinic C4, despite a similar focus on education and follow-ups, reports lower engagement rates (68.48% registration, 78.99% questionnaire completion), indicating that other clinic-specific or external factors may also play a role.

Clinics C3 and C6 report moderate engagement levels, with registration rates of 74.74% and 76.86% and questionnaire completion rates of 76.22% and 75.94%, respectively. Clinic C3's moderate performance may be attributed to its less aggressive approach in pursuing questionnaire completion, aiming to minimise patient burden and cognitive overload:

There's a series of questionnaires that are really, really helpful for us to measure. But I don’t put emphasis on necessity because I think otherwise, which discourages [patients] at times […] from a cognitive point of view, I think that the initial set-up phase, some of them say to me, can be quite exhausting in terms of – there's so much that you can input. And I do say do it over a number of days, a number of weeks, it doesn’t matter. C3.

This approach may lead to delays or neglect in completing registrations and questionnaires, impacting engagement levels.

Similarly, Clinic C6 adopts a systematic approach to onboarding, which includes an initial app introduction, followed by detailed guidance during the first week of group therapy sessions:

Before [patients] start [the group therapy sessions], we’ve normally spoken to them and told them that we would be sending them the invite to the app […]. But in terms of onboarding them to the concept of the app, that's something that we do in week one of our group session. We take them through each of the app's aspects in a bit of detail and explain where they can find things as well […]. C6.

However, this structured approach might delay the provision of immediate support, as noted by a patient:

That's what it felt like to start with: the app being passed without any support. It was only, I think, two weeks ago I actually had a phone call from the clinic regarding the therapy since I was using the app. I would have thought about four or five months now, and it's taken that amount of time for them actually to get in contact with me. A5.

#### Technical guidance

Clinics C5 and C7 implement a technical guidance approach to onboarding, focusing primarily on acquainting patients with LWCR functionalities and guiding them through the download and setup process. This method contrasts with other clinics integrating comprehensive patient education about the app's clinical relevance. For instance, clinic C7's strategy involves administrative staff offering the app during initial assessments:

[During initial assessments], the admin staff will often offer, ‘Would you like an app? We've got the living with the app. It's free because, at the moment for us, it is free, and we would like to sign you up. We need your email address. Are you happy and give your consent to do that?’ then they will often sign them up if they don't sign off at that point or they want to think about it, then we'll speak to them again at the end of their initial assessment. C7.

Despite employing similar technical approaches, clinics C5 and C7 exhibit varied performance in initial engagement metrics. Clinic C5 achieves moderate performance with registration rates of 83.85% and questionnaire completion rates of 85.74%. In contrast, Clinic C7 shows significantly lower performance, with registration rates of 64.38% and questionnaire completion rates of 71.29%. This discrepancy raises questions about the influence of clinical factors beyond the onboarding strategy itself.

Clinic C2, while less detailed about its onboarding strategy, registers top performance in both metrics. However, patient feedback indicates a significant communication gap regarding the app's purpose and usage, as described by patients A4 and A5:

[The onboarding] was a very quick whistle-stop tour. It might have just been that I wasn’t taking in things at that point. Maybe it was more in-depth. But I mean, again, it's quite self-explanatory when you go into it and work through it. A4.

Basically, I got a text saying, ‘Ring this phone number’. I rang the phone number, and this is basically just a secretary saying, ‘I’m going to send you a link to an app, and that's a Long COVID app, download it’. Nobody communicated with me about the app, the function of the app, or any treatment or intervention or anything. So, I downloaded the app, and that was it. […]. A5.

These accounts suggest that while C2 achieves high engagement metrics, the absence of comprehensive patient education might lead to confusion, potentially diminishing the app's perceived relevance and utility in patient care.

#### Key observations

Clinics implementing proactive and personalised follow-up strategies, like C1, achieve higher engagement levels. In contrast, clinics with a less urgent approach, like C3 and C6, experience moderate engagement, potentially impacting the app's perceived necessity and immediacy of support. The mixed results across C5, C7 and C2 indicate that technical guidance alone may be insufficient for optimal initial patient engagement. Clinic C5's moderate performance suggests that some patients may respond adequately to straightforward technical onboarding. However, the lower outcomes at clinic C7 and the critical feedback from C2 highlight the necessity for more comprehensive patient education to enhance understanding and foster better engagement.

##### Personnel involved in onboarding (who)

The personnel involved in the onboarding process vary across clinics, including clinically experienced staff such as clinicians, care coordinators and administrative staff. Although there is no distinct pattern correlating personnel type with clinic performance, top-performing clinics tend to employ clinically experienced staff for onboarding.

#### Clinically experienced staff

Clinics C1, C2, C3, C4 and C6 engage clinically experienced staff in onboarding, with some assigning care coordinators and others involving clinicians directly.

#### Care coordinators

Clinics C1 and C4 use care coordinators for onboarding. While not clinicians, these staff members bring clinical experience to the process. A clinician from C4 highlighted the importance of this approach:

We all agreed in our team that it shouldn’t be an admin person onboarding people onto the app; actually, it should be a clinical conversation that doesn’t necessarily need to be a qualified member of staff but should be somebody who comes from the rehab team themselves.C4.

Despite similar staffing, the performance outcomes vary: Clinic C1 achieves top performance in initial engagement, whereas Clinic C4 shows lower performance, indicating that other factors may influence these outcomes.

##### Clinicians

Clinics C2, C3 and C6, where clinicians directly manage the onboarding process, demonstrate top to moderate performance in initial engagement. Clinic C2, in particular, shifted to clinician-led onboarding to address delays associated with administrative processing:

We had very, very little admin, so sending a task to admin for onboarding was creating a completely unnecessary delay in a process that, in practice, took. It was quicker and easier just to do it ourselves, the onboarding.C2.

This change suggests that direct involvement by clinical staff can streamline processes and improve engagement metrics.

#### Administrative staff

Clinics C5 and C7 use administrative staff for onboarding, with varying degrees of success. Clinic C5 performs moderately well, while Clinic C7 is a LP. The lower performance at Clinic C7 may stem from the administrative staff's limited clinical background, potentially restricting the depth of patient onboarding discussions. Clinic C5 recognises this limitation and is considering involving care coordinators to enhance patient education:

At the moment, we’ve got an admin person doing [the onboarding], and they have a phone call, and they’ve got a little bit of an idea about what it is. But I think in time, to have the care coordinator doing [the onboarding], so somebody who's got a bit more of a clinical head so that they understand a bit more what long COVID is and how it might be affecting people. C5.

This potential shift reflects a broader recognition of the benefits that clinical insights can bring to initial patient engagement.

#### Key observations

The involvement of clinically experienced staff in onboarding is a common factor among top-performing clinics such as C1 and C2. In contrast, administrative-led onboarding might benefit from integrating more clinical expertise to enhance patient education and engagement.

##### Timing of onboarding (when)

The timing of the onboarding process varies across clinics. Some clinics begin onboarding to LWCR during the initial patient contact before the initial assessment, while others initiate onboarding during the initial consultation after the assessment. Generally, clinics adopting pre-assessment onboarding strategies report better initial patient engagement outcomes than those initiating post-assessment.

#### Pre-assessment onboarding

Clinics C1, C2, C4, C5 and C6 introduce LWCR early in the patient journey, immediately following a referral and before the initial assessment. Early engagement helps patients feel more involved and committed to their healthcare, contributing to top to moderate performance across these clinics. A clinician at C1 described the impact:

The fact that we can get [patients] onboarded on to the app [before their initial consultation] and get them to feel like they’re in – rather than just sitting at home for eight weeks waiting for an appointment. So, they feel engaged with[…] The number of calls that we get about, ‘I was referred, and I haven’t heard anything’ has almost vanished. Because we’re in contact with them almost straight away. C1.

This group includes three highest-performing clinics (C1, C2, C5). Clinics C2 and C5 shifted from post- to pre-assessment onboarding to mitigate administrative delays and improve efficiency. For example, a clinician from C5 explains:

We were enrolling people after we’d done the initial assessment, but that meant we collected a load of proms data from people on paper […] So, we had all those issues. So, what we’re doing now is asking people to join the app and complete the proms and symptom diary and demographics and all those bits ahead of their appointment. So, when they come for their first appointment, we can see the scores, so that speeds things up quite a bit for us. C5.

Clinic C4, despite also adopting a pre-assessment approach, shows lower performance, indicating that external factors may also play a significant role in influencing outcomes.

#### Post-assessment onboarding

Clinics C3 and C7 introduce LWCR during the initial consultation, following a more traditional in-person assessment process. A clinician from C7 outlines their approach:

I say to patients, come [to the clinic], and we'll do a 60 to 90 min assessment to see what your problems are; gives them a chance to tell me their narrative. They're story, validate that, and I value that because then I also find out what's been going on in the background. So, I want to use it as a kind of getting-to-know-you. C7.

Despite the potential benefits of this method, these clinics exhibit lower performance in both registration rates and questionnaire completion compared to clinics using pre-assessment onboarding. The delayed engagement might reduce the perceived relevance of the system to the patient's care journey, and the lack of urgency in completing system-based PROMs as part of the initial assessment may contribute to lower engagement levels.

#### Key observations

The timing of onboarding is a critical factor in patient engagement. Early onboarding (pre-assessment) tends to result in higher engagement levels, likely due to immediate involvement and reduced patient anxiety about the care process. In contrast, clinics employing post-assessment onboarding face challenges in motivating timely interaction with LWCR, ultimately affecting engagement scores. This suggests a clear benefit to integrating system interactions earlier in the patient journey to enhance engagement and satisfaction.

### Quantitative results: clinic interaction ongoing engagement metrics

This section summarises the quantitative findings on ongoing patient engagement in Tables 8 to 10. These tables detail clinic performance based on metrics such as average questionnaire completions and patient message rates within the first three months post-registration. We explore how clinics’ proactive and responsive communication strategies impact ongoing engagement. For detailed tables and further analysis, refer to the supplemental document.

**Table 8.** Clinic interaction ongoing engagement metrics.

Clinic #	Average questionnaires per patient		Average patient message per patient		Clinic message rate	
C1	38.64	TP	1.43	TP	88.26	TP
C2	35.34	TP	0.78	MP	31.05	MP
C3	31.12	MP	0.26	LP	10.59	LP
C4	34.19	MP	1.29	TP	75.89	TP
C5	31.90	MP	0.66	MP	35.63	MP
C6	29.94	MP	1.00	MP	32.10	MP
C7	27.37	LP	0.19	LP	13.54	LP

LP: lower performers; MP: moderate performers; TP: top performers.

**Table 9.** Descriptive statistics.

Statistics	Mean	Median	*SD*	Minimum	Maximum	Range	IQR	Q1	Q3
Average questionnaire per patient	32.64	31.90	3.73	27.37	38.64	11.27	5.4	29.94	35.34
Average patient message per patient	0.80	0.78	0.48	0.19	1.43	1.24	1.03	0.26	1.29
Clinic message rate (%)	41.00	32.10	29.82	10.59	88.86	77.67	62.35	13.54	75.89

IQR: interquartile range.

**Table 10.** Overall performance ranking and classification for clinic interaction ongoing engagement metrics.

Clinic	AQCP	APMP	Total	Average	Rank	Classification
C1	3	3	6	3	1	Top
C2	3	2	5	2.5	2	Top
C4	2	3	5	2.5	2	Top
C6	2	2	4	2	4	Moderate
C5	2	2	4	2	4	Moderate
C3	2	1	3	1.5	6	Moderate
C7	1	1	2	1.2	7	Lower

AQCP: average questionnaire completion per patient; APMP: average patient message per patient.

#### Overall performance ranking and classification

Clinics vary notably in their engagement metrics, with average questionnaires per patient ranging from 27.37 to 38.64, patient messages from 0.19 to 1.43 and clinic message rates from 10.59% to 88.26%. Within this framework, Clinic C1 stands out by leading in all categories, marking it as a TP. Conversely, Clinics C2 and C4, while also TPs, show a significant variance in clinic message rates, with C2 at a moderate level. Clinics at the lower spectrum, like C3 and C7, score minimally on patient messaging and clinic message rates, with C7 being the lowest in questionnaire completion. MPs like C5 and C6 exhibit middle-ground engagement metrics, maintaining consistent but unspectacular scores across the board.

### Comparative insights: clinical interaction and ongoing patient engagement

This section evaluates the relationship between clinic interaction efforts within LWCR and ongoing patient engagement metrics within 3 months post-registration. We compare quantitative clinic performance, such as average questionnaires completed per patient and average patient messages per patient, against qualitative feedback on clinic interaction strategies. Additionally, we examine clinic message rates to substantiate the relationship between clinic interactions and patient engagement outcomes.

#### Qualitative themes: clinic interaction strategies via LWCR

Two key themes were identified based on the thematic analysis of the clinics’ interaction strategies. They include:

1. Active interaction approaches.
2. Passive interaction approaches.

Clinic interaction strategies vary among clinics. Some employ active strategies involving frequent communication via LWCR, while others adopt passive approaches that depend on patient outreach. Our analysis reveals that clinics using active interaction strategies achieve higher performance ratings in ongoing patient engagement than those with passive approaches. This indicates that proactive and responsive patient management positively influences engagement outcomes.

#### Active interaction approaches

Clinics C1, C2, C4 and C6 actively engage patients through LWCR by monitoring patient data and proactively communicating via the app's messaging function. These efforts are reflected in their top to moderate clinic message rates (C1: 88.26%, C4: 75.89%, C6: 32.10% and C2: 31.05%). A clinician from C4 describes this proactive approach:

One particular patient had a dip towards the end of October, so I questioned her, and she said oh yes, I had a birthday party to organise, so I was organising for that particular week. I was doing more activity, so my fatigue levels went up […] So, it means that in terms of your time, you know you can use better spend it better looking on the app and contacting patients when need be. C4. (C1 appendix)

Such active engagement correlates with high averages in completed questionnaires and patient messages. For instance, C1 and C4, which have the highest clinic messaging rates, also report the highest average patient messages per patient at 1.43 and 1.29, respectively.

Moreover, patient feedback highlights the value of responsive communication:

If I’m feeling really low or unwell, I literally just go on [the app] and have a quick chat. Yeah, and then I also explain to them what my GP is doing next. Yeah, so it might take them a day or a day and a half to get back to me, but they do get back to me, and it's comforting to know that somebody's there. A12.

#### Role of intermediaries: care coordinators and support staff

Clinics within this group emphasised the crucial role of care coordinators in managing patient communication and sustaining engagement. By acting as intermediaries, care coordinators ensure efficient management of patient interactions, contributing to higher performance metrics. For instance, one of the clinicians at C1 noted:

Having [the care coordinator] in that kind of care-coordination role is really useful because she keeps an eye on things. And so, we rely on her to track who's using it and who's not. C1.

At Clinic C6, the introduction of care coordinators improved the management of the LWCR clinician dashboard, especially given a high patient load:

We’ve now got the care coordinators on board who will be checking [the dashboard] a couple of times a day […] I think the biggest thing is that we’ve got more capacity within the team to interact with the app and the patients […] Because before, we just didn’t have time to respond to the messages. C6.

Clinic C5 recognises the potential benefits of care coordinators and plans to integrate them to enhance patient engagement:

We’re recruiting a care coordinator to support us in the work […]. Maybe if we need to prompt people to do their proms, flag up to us anyone who's struggling so that we don’t have to have our heads in the data all the time, and we can be using [the care coordinators] to support us in what we’re doing. C5.

#### Passive interaction approaches

Clinics C3, C5 and C7, which adopt more passive interaction strategies, demonstrate lower ongoing patient engagement performance than clinics employing active approaches. This passive strategy typically involves patients self-monitoring and initiating contact as needed, potentially leading to decreased proactive patient engagement.

This approach is reflected in clinics’ moderate to lower message rates (C3: 10.59%, C7: 13.57%, C5: 35.63%), which correlate with lower ongoing patient engagement metrics, specifically average messages per patient (C7: 0.19, C3: 0.26, C5: 0.66). The notably low performance in Clinics C7 and C3 suggests that most patients do not communicate proactively or responsively with these clinics.

Operational challenges have forced Clinics C3 and C5 to adopt this hands-off approach due to high caseloads and limited hours:

I thought I would check every morning and see who's deteriorating and who's not and reach out. And I haven't because I can't. Especially with such large numbers on there, so I suppose I'm putting more ownership now on patients that if they you know need to reach out. C3.

Because of the numbers and because it was me on 11 h a week, I haven’t been able to go back to people. I’ve left it in their court to come back to me when they need help. And some of those people have been sitting around now for maybe six months, and I haven’t heard from them. C5.

Patient feedback from outside these clinics highlights the impact of this lack of clinic engagement. For example, one patient expressed:

I did actually think I might get to know if my symptoms were going up or down or whether they’d stagnated or whatever, but it doesn’t give you any sort of historic of how you’re doing yourself. I presume, obviously, the information is going somewhere, and someone's recording it. It's alright knowing this information, but if you know that someone is sort of – or you have a feeling that they’re not feeling very good, they’re feeling quite depressed, and they might do something, or someone's going down the wrong route, health-wise, I don’t know whether anybody would follow that up.

This feedback emphasises the significant consequences of limited clinic interaction on patient engagement with the LWCR system, suggesting that clinics reconsider their engagement strategies to support patient care effectively.

#### Key observations

Clinics employing proactive communication strategies, supported by the integration of care coordinators, consistently achieve higher engagement metrics than those with passive approaches. Active strategies facilitate timely responses and personalised patient support, fostering patient satisfaction and system utilisation. Conversely, passive strategy, characterised by self-monitoring and reliance on patient-initiated contact, is linked to lower engagement levels. These findings highlight clinics’ need to adopt more proactive and responsive interaction approaches to optimise patient engagement within the LWCR system.

## Discussion

As healthcare systems continue to navigate the complexities of long COVID, this study highlights the pivotal role of clinical practices in enhancing patient engagement with DHIs. Unlike previous studies, which have primarily focused on patient-related or technology-related factors,^13^ the current research uniquely explores the impact of clinical factors, specifically onboarding and interaction strategies, on patient engagement. The mixed-method comparison between quantitative clinic performance rankings and qualitative insights into onboarding and interaction practices revealed that higher-ranking clinics effectively implemented four main engagement strategies: pre-assessment onboarding, proactive communication, involvement of clinically experienced staff and comprehensive patient education. However, our findings suggest that the efficacy of these strategies is not solely dependent on their isolated implementation; instead, clinics that integrate multiple strategies tend to achieve higher and more consistent engagement levels. This highlights the necessity of a comprehensive approach in applying these strategies within clinical settings to optimise care for long COVID.

In the following sections, we discuss each strategy, examining its contributions to patient engagement and outlining how its integrated application can enhance the effectiveness of long-term COVID care.

### Pre-assessment onboarding

 Clinics implementing pre-assessment onboarding strategies achieve significantly higher initial patient engagement levels than those employing post-assessment methods. Specifically, we identified early patient education and the timely completion of baseline Patient-Reported Outcome Measures (PROMs) as key drivers of higher initial engagement metrics. This proactive approach facilitates immediate patient involvement, establishing active and sustained engagement throughout their care journey. Clinics that successfully implement this strategy motivate patients by demonstrating the direct impact of their early participation on subsequent clinical interactions and equip clinicians with essential baseline data for more personalised and effective initial consultations.

This finding is notable as it addresses a gap in the existing digital health literature; the direct impact of specific pre-assessment onboarding practices on patient engagement has not been extensively explored. Therefore, our study contributes new insights into how early engagement practices can significantly influence patient engagement. While pre-assessment onboarding has proven beneficial for enhancing initial patient engagement, it should not be viewed as a standalone strategy. To maximise effectiveness, this strategy must be integrated into a comprehensive engagement approach. This includes educating patients about the significance of their early contributions and explaining how these contributions directly enhance the quality of their care. Such integrative strategies are crucial for cultivating a sense of ownership and value among patients, ultimately leading to better engagement and care outcomes.

### Patient education and clinical staff involvement

 Clinics employing patient education strategies led by clinically experienced staff, such as care coordinators and clinicians, achieve higher patient engagement levels than those relying solely on technical guidance from administrative staff. Moreover, clinics that integrated early patient education within broader engagement strategies, particularly pre-assessment onboarding, achieved even greater initial engagement metrics. The nature and delivery of educational strategies varied across clinics, with sessions delivered either in person or remotely. Some clinics conducted one-on-one verbal sessions that explained the clinical relevance of the program and detailed the benefits of patient engagement for treatment outcomes. Others incorporated educational content into group therapy sessions using systematic presentations and screenshots to enhance understanding.

The use of clinically experienced staff for patient education proved significantly superior to that of administrative staff. Clinically experienced staff provided valuable insights into the clinical nuances of long COVID care and personalised guidance, enhancing patient understanding and engagement. This approach mirrors the successes observed in the psychosis recovery (A4i) study,^16^ where tailored educational materials and the involvement of informed personnel facilitated higher engagement rates.

The results from the A4i study^16^ and the current study highlight the significant benefit of clinically informed educational strategies in enhancing patient engagement within DHIs, particularly for complex chronic conditions like long COVID. We recommend that healthcare systems integrate these strategies as a fundamental component of their onboarding process, ensuring patients are equipped with tailored, clinically relevant information right from the beginning. This proactive approach is likely to improve patient engagement and outcomes significantly.

### Proactive (and responsive) clinic communication

Clinics utilising proactive communication strategies, such as initiating contact during onboarding and regular follow-ups via in-app messaging, achieve higher patient engagement levels than those with more passive approaches. Specifically, proactive interactions – including monitoring patient data entries and sending timely in-app messages – were linked to higher levels of patient registration, questionnaire completion and overall communication effectiveness. Such strategies enable clinicians to promptly address concerns and sustain continuous support, aligning with existing literature emphasising the importance of ongoing support systems in managing chronic conditions.^14–16^

We recommend healthcare providers encourage clinical staff to implement these proactive communication practices from the onset of the patient's journey. By doing so, they can ensure that patients consistently feel supported, heard and connected, thereby strengthening the patient–provider relationship and enhancing care outcomes.

#### The role of care coordinators

This study highlights the significant impact of care coordinators in enhancing patient engagement within the LWCR system. As crucial intermediaries, they maintain communication and support between patients and healthcare providers. Their proactive strategies, including regular check-ins and responsive communication, enable timely interactions and interventions, essential in clinics managing large patient volumes.

This role remains underexplored, with limited studies detailing the specific contributions of care coordinators in digital health systems. We recommend broader integration of care coordinators into patient management strategies to optimise patient engagement and operational efficiency. Training programs should focus on proactive communication and patient data analysis skills to equip care coordinators for effective real-time patient management. Future research should further explore the direct impacts of care coordination on patient outcomes to substantiate and refine these practices.

### Interdependencies between onboarding and interaction practices

The findings suggest that the effectiveness of onboarding and interaction practices is significantly enhanced when these practices are implemented as part of an integrated strategy rather than in isolation. Through the mixed-methods comparative analysis, we identified that clinics which successfully combined pre-assessment onboarding, proactive communication, comprehensive patient education and the involvement of clinically experienced staff achieved notably higher patient engagement levels. This interdependency suggests that these practices are not stand-alone interventions but mutually reinforcing elements of a cohesive patient engagement strategy.

For instance, Clinic C1, which exhibited the highest patient engagement metrics, effectively combined proactive follow-ups with early onboarding and extensive patient education. This integrated approach ensured that patients were initially engaged and actively involved in their care. In contrast, clinics that employed these practices independently or less consistently, such as Clinic C7, saw comparatively lower engagement, underscoring the importance of a holistic implementation.

These findings imply that healthcare providers aiming to enhance patient engagement in DHIs should prioritise the development of comprehensive strategies that incorporate multiple interdependent practices. Such an approach will yield more sustained and meaningful engagement, ultimately leading to better patient outcomes.

## Limitations

This research faced several limitations that should be taken into account when interpreting the results and considering future research directions. Ethical approval delays and high clinician workloads restricted our interviews to seven clinics. Of the over 200 patients approached, only 12 from six clinics participated, narrowing our data range. Additionally, only two clinics had overlapping clinician and patient interviews, which limited our comparative analysis within the same settings.

The quantitative data was primarily descriptive and aimed to identify best practice patterns by comparing clinic performances with qualitative insights. However, variability in clinic resources, staff availability, geographical contexts and patient demographics hindered our broader inferential conclusions. For instance, different resource levels between clinics could skew patient engagement outcomes. Including only seven clinics also challenged the reliability of our quartile-based classifications. We adjusted the analysis to reflect relative performance more accurately, enhancing reliability despite the small sample size.

## Conclusion

### Research contributions

This study extends the understanding of how clinic-led onboarding and interaction strategies influence patient engagement in DHIs, particularly in long COVID care. Through a mixed-methods approach, this research addresses a gap in the existing literature by emphasising the critical role of healthcare providers in patient engagement—an area often overshadowed by patient- and technology-centric factors. Specifically, the study identifies four key clinical practices – pre-assessment onboarding, proactive communication, comprehensive patient education and the involvement of clinically experienced staff – that collectively enhance patient engagement. These findings offer new conceptual insights for optimising DHIs in chronic care settings, with potential applications beyond long COVID. Additionally, this work lays the groundwork for future research to explore the broader implications of these strategies in various healthcare contexts.

### Practical implications

Practically, our findings provide actionable insights for healthcare providers and policymakers aiming to enhance the effectiveness of DHIs in clinical settings. Clinics can benefit from integrating pre-assessment onboarding to facilitate early patient involvement and leveraging clinically informed patient education to ensure that patients fully understand the benefits of their participation. Moreover, the critical role of care coordinators in maintaining ongoing patient engagement suggests that healthcare systems should invest in training and employing these professionals to manage patient interactions effectively. Implementing these strategies helps reduce the burden on healthcare resources and ensures continuous patient monitoring and support. While these strategies are particularly relevant to long COVID care, they can be adapted to other chronic care settings, offering a blueprint for improving overall care quality.

### Future work

Building on this study, future research should include a broader range of clinics to facilitate a more robust comparison of clinic practices and patient engagement across varied clinical settings. It is crucial to investigate the interactive effects of the identified engagement strategies, focusing on their optimal collective implementation to maximise patient engagement. Additionally, longitudinal studies are essential to determine the long-term impacts of these strategies on patient engagement and health outcomes. Such studies should track engagement over extended periods to assess the sustainability of the effects observed. Moreover, given the positive role of care coordinators demonstrated in this study, further exploration of their specific contributions and effectiveness in various clinical settings is warranted, particularly in comparison to direct clinician involvement.

## Supplemental Material

sj-docx-1-dhj-10.1177_20552076241294101 - Supplemental material for Comparative insights into clinic onboarding and interaction practices for patient engagement in long COVID digital health care Supplemental material, sj-docx-1-dhj-10.1177_20552076241294101 for Comparative insights into clinic onboarding and interaction practices for patient engagement in long COVID digital health care by Hadiza Ismaila, Ann Blandford, David Sunkersing, Fiona Stevenson and Henry Goodfellow in DIGITAL HEALTH

sj-docx-2-dhj-10.1177_20552076241294101 - Supplemental material for Comparative insights into clinic onboarding and interaction practices for patient engagement in long COVID digital health care Supplemental material, sj-docx-2-dhj-10.1177_20552076241294101 for Comparative insights into clinic onboarding and interaction practices for patient engagement in long COVID digital health care by Hadiza Ismaila, Ann Blandford, David Sunkersing, Fiona Stevenson and Henry Goodfellow in DIGITAL HEALTH

sj-docx-3-dhj-10.1177_20552076241294101 - Supplemental material for Comparative insights into clinic onboarding and interaction practices for patient engagement in long COVID digital health care Supplemental material, sj-docx-3-dhj-10.1177_20552076241294101 for Comparative insights into clinic onboarding and interaction practices for patient engagement in long COVID digital health care by Hadiza Ismaila, Ann Blandford, David Sunkersing, Fiona Stevenson and Henry Goodfellow in DIGITAL HEALTH
