# Supplementary material for: Comparative insights into clinic onboarding and interaction practices for patient engagement in long COVID digital health care
Source: Digit Health. 2024 Nov 26;10:20552076241294101. doi: 10.1177/20552076241294101 (PMC11590151; doi:10.1177/20552076241294101)
Supplement: sj-docx-1-dhj-10.1177_20552076241294101 - Supplemental material for Comparative insights into clinic onboarding and interaction practices for patient engagement in long COVID digital health care [file sj-docx-1-dhj-10.1177_20552076241294101.docx]

Comparative Insights into Clinic Onboarding and Interaction Practices for Patient Engagement in Long COVID Digital Health Care: Supplemental Content

## Quantitative Results

### Onboarding Initial Engagement Metrics

This section presents descriptive quantitative findings from our initial uptake patient registration analysis and first engagement activities, such as questionnaire completion within the first month post-registration.

#### Patient Registration: Registration Rate

The registration rate metric measures the proportion of patients who register on the platform after receiving an invitation. This measure is crucial as it indicates the effectiveness of the onboarding process in converting invited patients to registered users.

Table 1: Patient Registration Rates by Clinic

| Clinic # | Patients Invited | Patients registered | Registration Rate % | Rank | Classification |
| --- | --- | --- | --- | --- | --- |
| C1 | 320 | 292 | 91.25 | 1 | Top Performers |
| C2 | 792 | 708 | 89.39 | 2 | Top Performers |
| C5 | 1350 | 1132 | 83.86 | 3 | Moderate Performers |
| C6 | 604 | 464 | 76.82 | 4 | Moderate Performers |
| C3 | 689 | 515 | 74.75 | 5 | Moderate Performers |
| C4 | 184 | 126 | 68.48 | 6 | Lower Performers |
| C7 | 160 | 103 | 64.38 | 7 | Lower Performers |

Table 2: Descriptive Statistics

| **Statistics %** | **Mean** | **Median** | **SD** | **Minimum** | **Maximum** | **Range** | **IQR** | **Q1** | **Q3** |
| --- | --- | --- | --- | --- | --- | --- | --- | --- | --- |
| **Registration Rate** | 78.42 | 76.82 | 9.46 | 64.38 | 91.25 | 26.88 | 20.92 | 68.48 | 89.39 |

##### Descriptive Statistics Summary

The mean registration rate across the seven clinics is 78.42%, with a standard deviation (SD) of 9.46%, indicating a moderate variability among clinics. The range of registration rates is 26.88%, with an interquartile (IQR) range of 20.92%, reflecting a substantial spread, particularly among the central half of the clinics. The median registration rate is 76.82%, slightly below the mean, suggesting a slight skew towards lower rates. A boxplot analysis [shows the median's closer proximity to the first quartile, indicating a concentration of lower registration rates among some clinics.


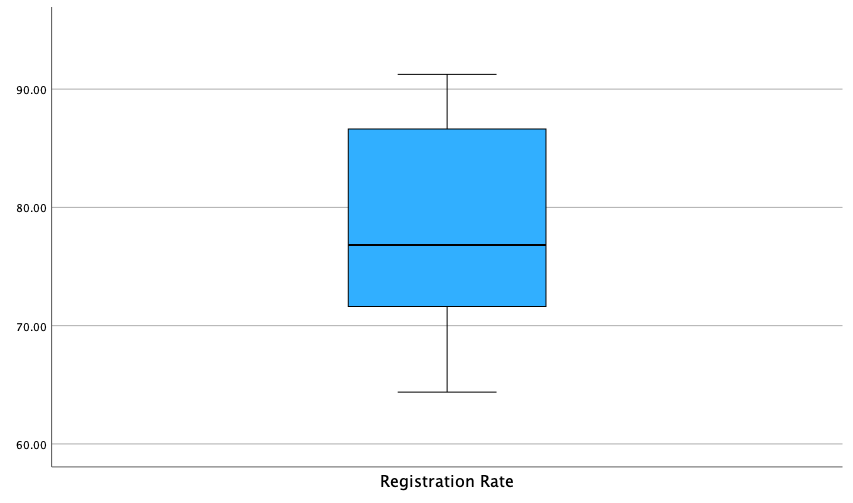


Figure 1: Boxplot Registration Rate

##### Performance Ranking and Classification

Given the proximity of Q3 and Q1 to their respective extremes, with Q3 near the maximum and Q1 closer to the minimum, the original classification thresholds remain appropriate. These thresholds ensure that the categories meaningfully reflect the actual performance dispersion among the clinics:

- **Top Performers:** Clinics C1 (91.25%) and C2 (89.39%), both of whose registration rates are at or above Q3 (89.39%), demonstrating exceptionally high registration rates that set benchmarks within the cohort.
- **Moderate Performers:** Clinic C5 (83.86%) nearly approaches the top performers, standing out with a registration rate considerably higher than the median but not reaching the top tier. Clinic C6 (76.82%) and Clinic C3 (74.75%) show adequate engagement, aligning close to the median (76.82%).
- **Lower Performers:** Clinic C4 (68.48%) and Clinic C7 (64.38%) are at or below the first quartile (Q1: 71.62%). Their considerably lower registration rates indicate potential patient onboarding challenges, highlighting improvement areas.

#### Questionnaire Completion: Questionnaire Completion Rate

The questionnaire completion rate measures the percentage of patients who complete at least one questionnaire within the first month of registration. This metric is crucial for assessing initial patient engagement and responsiveness to the onboarding process.

Table 3: Questionnaire Completion Rate by Clinic

| **Clinic #** | **Patients Completed Questionnaire** | **Patients Registered** | **Questionnaire Completion Rate** | **Rank** | **Performance Classification** |
| --- | --- | --- | --- | --- | --- |
| C2 | 620 | 689 | 89.96% | 1 | Top Performers |
| C1 | 242 | 276 | 87.68% | 2 | Top Performers |
| C5 | 920 | 1073 | 85.74% | 3 | Top Performers |
| C4 | 94 | 119 | 78.99% | 4 | Moderate Performers |
| C3 | 375 | 492 | 76.22% | 5 | Moderate Performers |
| C6 | 344 | 453 | 75.94% | 6 | Moderate Performers |
| C7 | 72 | 101 | 71.29% | 7 | Lower Performer |

Table 4: Descriptive Statistics

| **Statistics (%)** | **Mean** | **Median** | **SD** | **Minimum** | **Maximum** | **Range** | **IQR** | **Q1** | **Q3** |
| --- | --- | --- | --- | --- | --- | --- | --- | --- | --- |
| **Questionnaire Completion Rate** | 80.83 | 78.99 | 7.00 | 71.29 | 89.96 | 18.70 | 11.74 | 75.94 | 87.68 |

##### Descriptive Statistics Summary

The mean questionnaire completion rate within the first month of registration is 80.83%, with an SD of 7.00%, indicating moderate variability among the clinics. The range of 18.70% and an IQR of 11.74% suggest a moderate spread in rates. The median completion rate of 78.99%, only about 2.28% lower than the mean, indicates that the data distribution is relatively balanced with a slight inclination towards lower rates.

**
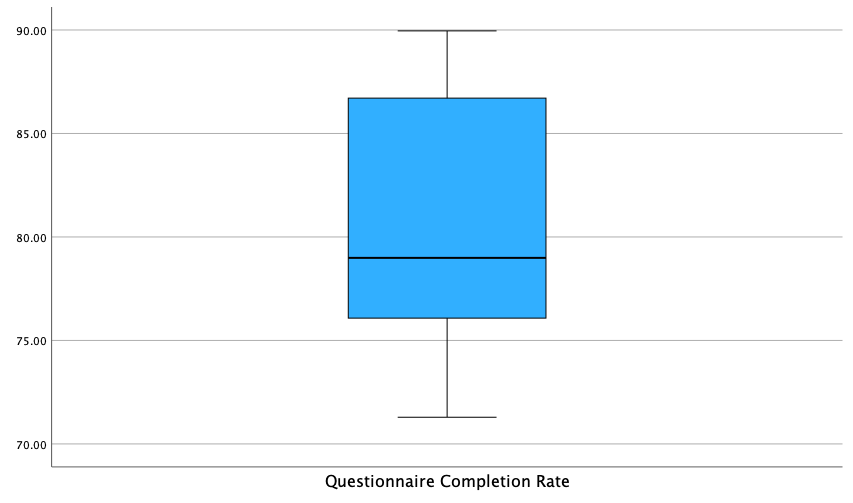
**

Figure 2: Boxplot Questionnaire Completion Rate

##### Performance Ranking and Classification

Clinic C5's questionnaire completion rate of 85.74%, while below Q3 (87.68%), substantially exceeds the median (78.99%) and closely approaches top performers. Given its performance and considering the narrow gap between the median and Q1, we adjusted the classification boundaries to reflect nuanced differences better:

- **Top Performers:** Clinics with rates significantly above the median (and near or above Q3). This category includes Clinics C2 (89.96%), C1 (87.68%), and C5 (85.74%), each demonstrating superior initial engagement.
- **Moderate Performers:** Clinics with completion rates near the median (and at or above Q1). This group includes Clinics C4 (78.99%), which matches the median, and Clinics C3 (76.22%) and C6 (75.94%), all reflecting modest engagement levels.
- **Lower Performers:** Clinics with completion rates relatively significantly lower than the median (and below Q1). This includes Clinic C7 (71.29%), indicating a performance level that falls below the cohort's average and highlights areas of improvement.

#### Questionnaire Completion: Initial Questionnaire Completion Speed

The initial questionnaire completion speed measures the average days it takes newly registered patients to complete their first questionnaire. This metric is crucial because it assesses the efficiency of the onboarding process and the immediacy of patient engagement following registration.

Table 5: Initial Questionnaire Completion Speed by Clinic

| Clinic # | Average Days to Questionnaire Completion | Rank | Classification |
| --- | --- | --- | --- |
| C1 | 1.14 | 1 | Top Performers |
| C2 | 1.56 | 2 | Moderate Performers |
| C7 | 1.68 | 3 | Moderate Performers |
| C5 | 1.80 | 4 | Moderate Performers |
| C6 | 2.08 | 5 | Moderate Performers |
| C3 | 2.23 | 6 | Lower Performers |
| C4 | 2.28 | 7 | Lower Performers |

Table 6: Descriptive Statistics

| **Statistics (Days)** | **Mean** | **Median** | **SD** | **Minimum** | **Maximum** | **Range** | **IQR** | **Q1** | **Q3** |
| --- | --- | --- | --- | --- | --- | --- | --- | --- | --- |
| **Questionnaire Completion Speed** | 1.82 | 1.80 | 0.40 | 1.14 | 2.28 | 1.14 | 0.67 | 1.56 | 2.23 |

##### Descriptive Statistics Summary

The average time it takes for patients to complete the initial questionnaire is 1.82 days, with a standard deviation of 0.40 days. This indicates low variability and a generally efficient initial patient engagement process across the clinics. The range of completion times is narrow, spanning only 1.14 days from a minimum of 1.14 days in Clinic C1 to a maximum of 2.28 days in Clinic C4. The median completion time is 1.80 days, closely aligning with the mean, suggesting a relatively balanced distribution of completion times across clinics. An IQR of 0.67 days further confirms the limited variability, showing that the middle 50% of clinics have completion times tightly grouped around the median.


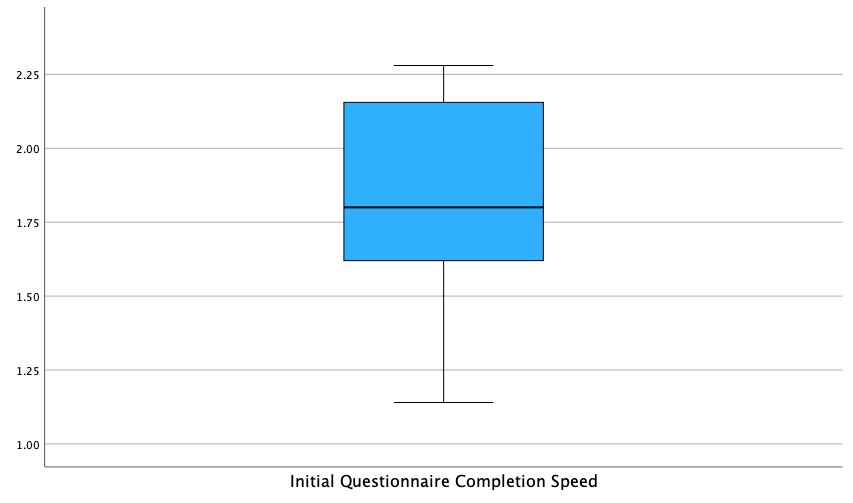


Figure 3: Boxplot Initial Questionnaire Completion Speed

##### Performance Ranking and Classification

Given the proximity of Q1 (1.56 days) to the median (1.80 days), we refined the classification boundaries for a more nuanced interpretation:

- **Top Performers:** Clinics with an average completion time below Q1 (1.56 days). This exclusively includes Clinic C1 (1.14 days), demonstrating exceptional efficiency and outperforming its counterparts.
- **Moderate Performers:** Clinics with completion times close to the central values (at or above Q1 but below Q3 (2.23 days)). This group comprises clinics C2 (1.56 days), C7 (1.68 days), C5 (1.80 days), and C6 (2.08), showcasing moderate efficiency.
- **Lower Performers:** Clinics with times at or above Q3, including C3 (2.23 days) and C4 (2.28 days). While these clinics have the slowest completion times, it’s essential to recognise that their performance does not drastically lag behind the moderate performers.

#### Comparing Questionnaire Completion Rates and Speed

Clinic C1 is a top performer in both questionnaire completion rate and speed, demonstrating superior efficiency and engagement. Conversely, Clinics C2 and C5, while top performers in completion rates, rank as moderate in speed, indicating that while many patients complete questionnaires, they do so at a less-than-optimal pace. Clinic C6 maintains a consistent moderate rank in both metrics, showing uniform but average performance. Clinics C3 and C4 have moderate completion rates but are slower, indicating less efficient processes. Notably, Clinic C7, despite a moderate completion speed, shows the lowest completion rate, suggesting that quicker processing does not necessarily correlate with higher engagement levels.

### Ongoing Engagement within the first three months of Registration

#### Average Questionnaire per Patient

The average questionnaire per patient metric measures the average number of questionnaires each participating patient completes, providing insight into how engaged and active they are in reporting their health status.

Table 7: Average Questionnaires per Patient by Clinic

| **Clinic #** | **Questionnaires Completed** | **Patients Completed** | **Average per Patient** | **Rank** | **Classification** |
| --- | --- | --- | --- | --- | --- |
| C1 | 8695 | 225 | 38.64 | 1 | Top Performers |
| C2 | 19722 | 558 | 35.34 | 2 | Top Performers |
| C4 | 3180 | 93 | 34.19 | 3 | Moderate Performers |
| C5 | 26857 | 842 | 31.90 | 4 | Moderate Performers |
| C3 | 11016 | 354 | 31.12 | 5 | Moderate Performers |
| C6 | 9551 | 319 | 29.94 | 6 | Moderate Performers |
| C7 | 1861 | 68 | 27.37 | 7 | Lower Performers |

Table 8: Descriptive Statistics

| **Statistics** | **Mean** | **Median** | **SD** | **Minimum** | **Maximum** | **Range** | **IQR** | **Q1** | **Q3** |
| --- | --- | --- | --- | --- | --- | --- | --- | --- | --- |
| **Average Questionnaire Per Patient** | 32.64 | 31.90 | 3.73 | 27.37 | 38.64 | 11.27 | 5.4 | 29.94 | 35.34 |

##### Descriptive Statistics Summary

The mean number of questionnaires completed per patient across clinics is 32.64, with a standard deviation of 3.73, indicating moderate variability. The distribution shows a range of 11.27 between the minimum (27.37) and maximum (38.64) averages. The median of 31.90, slightly lower than the mean, suggests a fairly symmetric distribution with a mild skew towards lower numbers of questionnaires completed per patient.

**
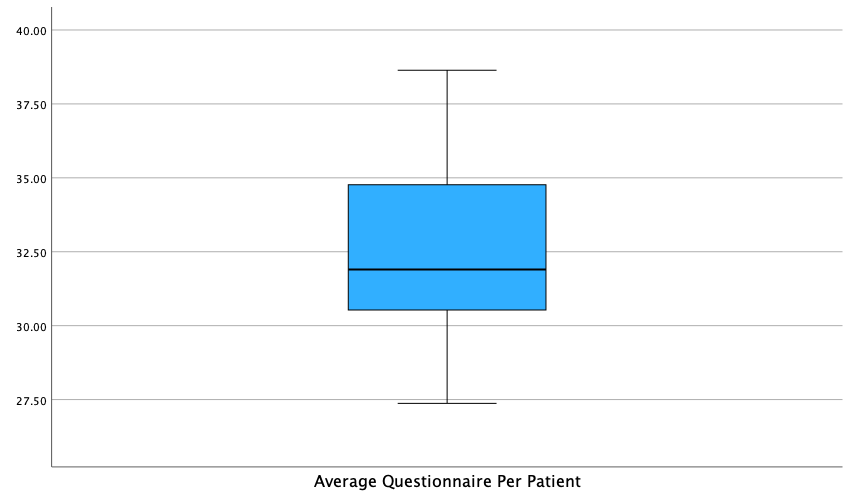
**

Figure 4: Average Questionnaire Per Patient

##### Performance Ranking and Classification

Table: Performance Classification Based on Average Questionnaires per Patient

Given the proximity of Q3 (35.34) to the maximum (38.64) and Q1 (29.94) being closer to the median (31.90) than the minimum (27.37), a slight adjustment in the classification boundary is warranted. We set the lower performers’ boundary below Q1 to more accurately reflect distinct performance levels. Clinic C6, exactly at Q1, is classified as a moderate performer, while clinic C7, below Q1, is classified as a lower performer.

- **Top Performers:** Clinics C1 (38.64) and C2 (35.34), both at or above Q3, demonstrate relatively high engagement.
- **Moderate Performers:** Clinics C4 (34.19), C5 (31.90), and C3 (31.12), ranging from below Q3 to at or above Q1, show average to above-average engagement.
- **Lower Performers:** Clinics C6 (29.94) and C7(27.37), positioned at and below Q1, indicate below-average performance.

##### Performance Ranking and Classification

Given Q3’s (C4, 33.04%) closer proximity to the mid-range values, including the median, than the maximum (C1, 44.13%), we have adjusted the classification boundaries as follows:

- **Top Performers:** Clinics with patient message rates above Q3 (33.04%). This includes only Clinic C1 (44.13%), showcasing the highest engagement in patient communication.
- **Moderate Performers:** Clinics with patient message rates at Q3 and above Q1 (10.42 to 33.04%). This group includes Clinic C4 (33.04%), C6 (27.90%), C2 (24.18%), and C5 (21.42%), reflecting modest engagement levels relative to most counterparts.
- **Lower Performers:** Clinics with patient message rates at or below Q1 (10.42%). This category includes Clinics C7 (10.42%) and C3 (9.69%), indicating room for improvement.

#### Clinic Message Rate

Clinic message rate measures the proportion of patients who receive messages from the clinic. This metric reflects the clinic's proactiveness in initiating contact and maintaining communication.

Table 9: Clinic Message Rate

| **Clinic #** | **Patients Received Messages** | **Patients Registered** | **Clinic Message Rate %** | **Ranking** | **Classification** |
| --- | --- | --- | --- | --- | --- |
| C1 | 218 | 247 |  | 1 | Top Performers |
| C4 | 85 | 112 | 75.89 | 2 | Top Performers |
| C5 | 341 | 957 | 35.63 | 3 | Moderate Performers |
| C6 | 130 | 405 | 32.10 | 4 | Moderate Performers |
| C2 | 190 | 612 | 31.05 | 5 | Moderate Performers |
| C7 | 13 | 96 | 13.54 | 6 | Lower Performers |
| C3 | 47 | 444 | 10.59 | 7 | Lower Performers |

Table 10: Descriptive Statistics

| **Statistics %** | **Mean** | **Median** | **SD** | **Minimum** | **Maximum** | **Range** | **IQR** | **Q1** | **Q3** |
| --- | --- | --- | --- | --- | --- | --- | --- | --- | --- |
| **Clinic Message Rate** | 41.00 | 32.10 | 29.82 | 10.59 | 88.86 | 77.67 | 62.35 | 13.54 | 75.89 |

##### Descriptive Statistics Summary

The average clinic message rate is 41.00%, with a high standard deviation of 29.82%, indicating significant clinic variability. The wide range, 77.67%, and a large IQR of 62.35 further emphasise clinic performance disparities. The median rate of 32.10% is substantially lower than the mean, indicating a distribution skewed towards lower message rates.

**
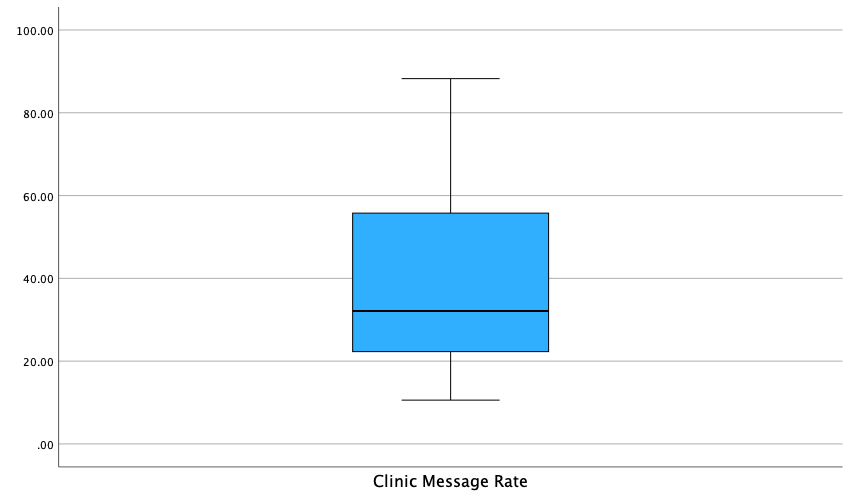
**

Figure 5: Clinic Message Rate

##### Performance Ranking and Classification

- **Top Performers:** Clinics with message rates at and above Q3 (75.89%). This includes Clinic C1 (88.26%)and C4 (75.89%), which exhibit exceptional interaction efforts.
- **Moderate Performers**: Clinics with message rates between Q3 and Q1. This includes Clinics C5 (35.63%), C6(32.10%), and C2(31.05%), showing average engagement performance.
- **Lower Performers:** Clinics with message rates at Q1 or below. This group includes Clinics C7 (13.54%) and C3 (10.59%), suggesting significant room for improvement in their messaging strategies.
