# Supplementary material for: Comparative insights into clinic onboarding and interaction practices for patient engagement in long COVID digital health care
Source: Digit Health. 2024 Nov 26;10:20552076241294101. doi: 10.1177/20552076241294101 (PMC11590151; doi:10.1177/20552076241294101)
Supplement: sj-docx-3-dhj-10.1177_20552076241294101 - Supplemental material for Comparative insights into clinic onboarding and interaction practices for patient engagement in long COVID digital health care [file sj-docx-3-dhj-10.1177_20552076241294101.docx]

DHJ-24-0631: Supplemental Content: Questionnaires Included in the LWCR Program

| **Questionnaire** | **Measures** | **More Information (example: some are available via different sources)** | **Number of questions** |
| --- | --- | --- | --- |
| MRC Dyspnoea | Degree of breathlessness related to activity. | <https://www.pcrs-uk.org/mrc-dyspnoea-scale> | 1 |
| Dyspnoea-12 | Overall score of breathlessness severity. | <https://www.ncbi.nlm.nih.gov/pmc/articles/PMC3035488/>  Also <https://toolkit.severeasthma.org.au/wp-content/uploads/sites/2/2018/03/Dyspnoea12Questionnaire.pdf> | 12 |
| Covid Recovery |  | Not validated. Question was: “Which of the following phrases best describes how you are getting on?” with five possible responses ranging from “I cannot do any of the normal activities that I could do before Covid” to “I can do all the normal activities that I could do before Covid”. | 1 |
| Functional Assessment of Chronic  Illness Therapy - Fatigue (FACIT-F) | Self-reported fatigue and its impact upon daily activities and function. | https://www.facit.org/measures/facit-f | 13 |
| Generalized Anxiety Disorder scale  (GAD-7) | Screening tool and severity measure for anxiety. | https://patient.info/doctor/generalised-anxiety-disorder-assessment-gad-7 | 7 |
| Work and Social Adjustment  scale (WSAS) | Measures perceived ability to work and engage with others. | https://greenspacehealth.com/en-us/work-and-social-functioning-wsas/ | 6 |
| EQ-5D-5L | Measures key experiences of health from different perspectives. | https://euroqol.org/information-and-support/euroqol-instruments/eq-5d-5l/ | 6 |
| Perceived Deficits Questionnaire, 5 item version (PDQ-5) | Measures the degree to which individuals perceive themselves as experiencing cognitive difficulties (“brain fog”). | https://eprovide.mapi-trust.org/instruments/perceived-deficits-questionnaire | 5 |
| Demographic | Records basic demographic information. | Year of birth, gender, ethnicity, highest level of educational achievement, postcode. | 5 |
| Patient Health Questionnaire  depression scale (PHQ-8) | A diagnostic and severity measure for current depressive disorders. | https://www.ncbi.nlm.nih.gov/pmc/articles/PMC3035488/ | 8 |
| Health Service Use | Patient-reported use of health services and working days list in preceding four weeks | Hospital outpatient appointments in the last 4 weeks, GP appointments in the last 4 weeks, Psychologist appts in the last 4 weeks, physiotherapy ditto, days off work (sick) ditto, as hospital in-patient ditto. | 6 |
